# Peritoneal carcinomatosis, an unusual and only site of metastasis from lung adenocarcinoma

**DOI:** 10.11604/pamj.2016.23.60.8910

**Published:** 2016-02-29

**Authors:** Kloub Hanane, Benhmida Salma, Bellahammou Khadija, Elghissassi Ibrahim, Boutayeb Saber, M'rabti Hind, Errihani Hassan

**Affiliations:** 1 Department of Medical Oncology, National Institute of Oncology, Rabat, Morocco

**Keywords:** Lung cancer, peritoneal carcinomatosis, chemotherapy

## Abstract

Isolated peritoneal metastases of lung adenocarcinoma are very rare, even exceptional, occurring most often in the context of a multi-metastatic disease. This report presents a rare clinical case of isolated peritoneal metastasis from lung adenocarcinoma. We report a 56-year-old male who was monitored for lung adenocarcinoma whose evolution has been marked by an isolated metastatic recurrence in the peritoneum objectified by an abdominal-pelvic computed tomography (CT) and confirmed by a laparoscopy with abiopsy of the peritoneal nodules. The patient had received palliative chemotherapy with gemcitabine, cisplatin and bevacizumab. The evolution was marked by a progressive deterioration of the general condition and death two months after the third treatment cycle. Peritoneal carcinomatosis from lung adenocarcinoma is a very rare event, and is often associated with a poor prognosis.

## Introduction

Lung cancer is the world's most frequently diagnosed cancer (1.82 million) and the leading cause of cancer related death (1.6 million deaths) [1]. Conventional metastatic sites are the liver, adrenal glands, the brain and bone. Isolated peritoneal metastasis is a very rare event. This report presents a rare clinical case of isolated peritoneal metastasis from lung adenocarcinoma.

## Patient and observation

**Figure 1:**
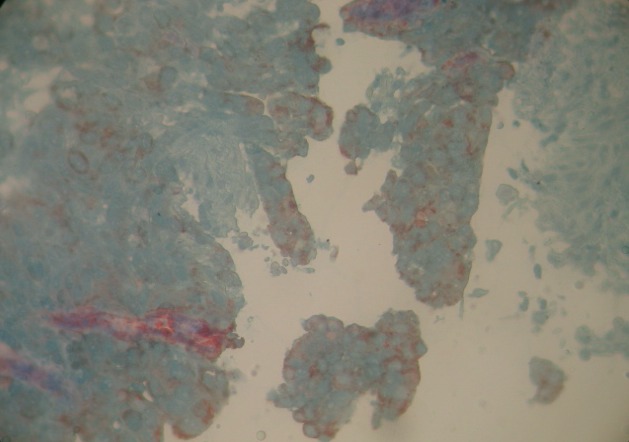
Biopsy of the peritoneal nodules shows positive staining CK7 on immunohistochemistry

**Figure 2:**
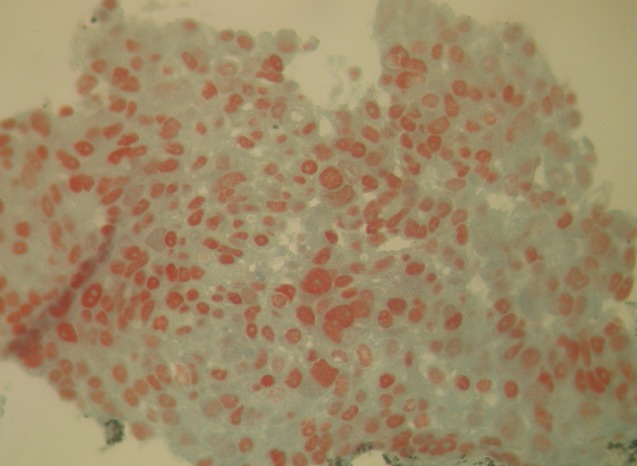
Biopsy of the peritoneal nodules shows positive staining TTF1 on immunohistochemistry

**Figure 3:**
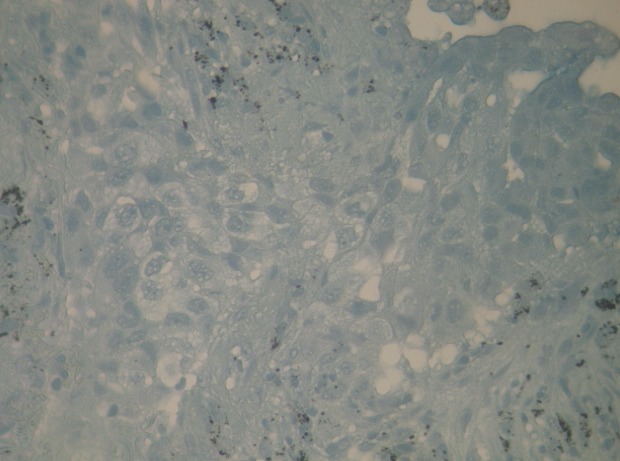
Biopsy of the peritoneal nodules: CK20 negative on immunohistochemistry

A 56-year old male, with history of chronic smoking, was monitored from 2011 for a lung adenocarcinoma, initially classified as stage IIIA (pT2N3M0). The patient underwent a right upper lobectomy with lymph node dissection in January 2011. Adjuvant chemotherapy with paclitaxel and carboplatin was administered in 4 cycles until June 2011. In March 2012, he had abdominal pain. The abdominal-pelvic ultrasound showed small amounts of ascites. The hepatitis B and C serologies were normal, liver function tests and blood counts were normal. The chest and abdominal-pelvic computed tomography (CT) showed the presence of a moderate amount of ascites without obvious signs of lung relapse or abdominal visceral disease. Bone scan and brain CT were both normal. An ultrasound-guided puncture was attempted twice without success. The patient underwent exploratory laparoscopy which showed nodules of peritoneal carcinomatosis with a moderate amount of ascites. A biopsy of the peritoneal nodules was performed. The histological examination revealed adenocarcinoma. The immunohistochemistry showed that the carcinoma cells were positive for cytokeratin 7 (CK7) (Figure 1) and thyroid transcription factor-1 (TT1) (Figure 2) and negative for cytokeratin 20 (CK20) (Figure 3). Those immunohistological findings suggested the tumor to be metastatic from the primary lung adenocarcinoma. Genotyping of epidermal growth factor receptor (EGFR) exons 18 to 21 did not show any detectable mutation. The patient underwent palliative chemotherapy following the gemcitabine, cisplatin and bevacizumab protocol. The evolution was marked by a progressive deterioration of the general condition and death two months after the third treatment cycle.

## Discussion

Preferential metastases sites for lung cancer are the liver, adrenal glands, the brain and bone. While abdominal visceral metastases have been reported in the context of lung cancer [2–4], isolated peritoneal metastasis is a very rare event, most often described in old autopsy series [5]. It can occur in the context of a multi-organ metastasis and is only exceptionally indicative of a primary lung tumor. Satoh et al reviewed the observations of 1041 patients treated consecutively between 1976 and 2001; 12 patients (1.2%) developed peritoneal involvement over the course of the disease. Six and nine of the twelve patients had abdominal visceral metastases and pleural metastasis, respectively [6]. The frequency of the peritoneal metastasis was higher in the case of adenocarcinoma and large cell carcinoma. Other cases of peritoneal carcinomatosis of lung cancer have been reported in the literature but the majority of patients had advanced disease with multiple metastatic sites [7]. The tumors that most often accompany peritoneal disease are those of the ovary and the gastrointestinal tract [8]. Three implantation mechanisms of the tumor cells on the peritoneum have been described in the literature [9]; by contiguity expansion secondary to the involvement of the serosa and its supersession by the tumor resulting in exfoliation of tumor cells in the peritoneal cavity, where they are likely to take hold; iatrogenic expansion which may be secondary to the direct trauma of the tumor during its removal from where tumor cells circulate in the veins and lymph nodes severed during removal of the tumor. Systemic expansion is rare and can occur for a number of digestive and non-digestive cancers but at an advanced stage of development (breast, lungs, etc.). However, the mechanism of the isolated peritoneal dissemination of a lung cancer has not been studied [6]. Clinically, peritoneal carcinomatosis is usually asymptomatic at the early stage, making its detection less likely. In recent years, with the development and availability of new imaging techniques such as CT and PET scans, peritoneal carcinomatosis can be diagnosed more easily and accurately. In our case the diagnosis was confirmed by a diagnostic laparoscopy with biopsy of the peritoneal nodules. The immunohistochemistry usually comprises CK5/6, TTF1, CK7 and CK20. In our case, the immunophynotype of CK7 positive, CK20 negative and TTF1 positive makes the primitive nature of the lung cancer almost certain [10]. Despite recent medical and surgical advances, the treatment of peritoneal carcinomatosis is primarily palliative and the survival median is no more than two months [6, 7].

## Conclusion

Peritoneal carcinomatosis from lung cancer remains a rare event. This diagnosis should always be considered for the occurrence of unexplained abdominal symptoms, especially in a patient already treated for lung cancer.
